# Reappraisal of Grading in Intestinal-Type Sinonasal Adenocarcinoma: Tumor Budding as an Independent Prognostic Parameter

**DOI:** 10.1007/s12105-022-01410-3

**Published:** 2022-01-11

**Authors:** Christian M. Meerwein, Muriel D. Brada, Michael B. Soyka, David Holzmann, Niels J. Rupp

**Affiliations:** 1 Department of Otorhinolaryngology, Head & Neck Surgery, University Hospital of Zurich and University of Zurich, Frauenklinikstrasse 24, 8091 Zurich, Switzerland; 2 Department of Molecular Pathology, University Hospital of Zurich and University of Zurich, Zurich, Switzerland; 3 Faculty of Medicine, University of Zurich, Zurich, Switzerland

**Keywords:** Paranasal sinus neoplasms, Adenocarcinoma, Colorectal neoplasms, Epithelial–mesenchymal transition, Colorectal neoplasms, Skull base

## Abstract

Since sinonasal intestinal-type adenocarcinomas (ITAC) show resemblance to colorectal adenocarcinomas, we aimed to investigate novel prognostic factors of outcome, with particular focus on the role of tumor budding (TB). Retrospective clinico-pathological single-institution study on consecutive ITAC patients between 1996 and 2020. Histopathological parameters including conventional subtypes and TB features (low, intermediate, high) were evaluated with the aid of pancytokeratin (AE1/AE3) immunohistochemical staining. Parameters were correlated to clinical data and outcome. A total of 31 ITAC patients were included. Overall, 19/31 patients (61.3%) presented with stage III/IV disease. Presence of lymph node or distant metastases was rare (1/31 patient, 3.2%). Treatment protocols consisted of tumor resection in 30/31 patients (96.8%) and primary radiochemotherapy in 1/31 patient (3.2%). Adjuvant radiation therapy was conducted in 20/30 surgically treated patients (66.7%). The 3- and 5-year overall survival (OS) was 83.9% and 78.3% and the 3- and 5-years disease-specific survival (DSS) 83.7% % and 78.5%, respectively. The presence of intermediate/high TB (defined as ≥ 5 buds) was associated with both, worse DSS (log rank p = 0.03) and OS (log rank p = 0.006). No patient with low TB revealed progressive disease or died of the disease. No association between TB and tumor stage or conventional tumor subtype was found. Tumor budding seems to be an independent prognostic factor of worse outcome in ITAC.

## Introduction

Intestinal-type sinonasal adenocarcinoma (ITAC) is a rare epithelial sinonasal tract malignancy and accounts for approximately 8–25% of all sinonasal cancers [1]. Thereby, an incidence of approximately 0.7–1.4 cases/100,000/year was reported, with a higher proportion in Europe, compared to the United States [2, 3]. Especially for ethmoidal ITACs, previous studies revealed a strong predominance for men (male: female ratio = 21: 1), mainly due to an occupational exposition to known carcinogens, such as wood dust (relative risk 29.4) and leather dust [3–6]. In contrary, other carcinogens, such as asbestos, nickel/chrome or formaldehyde were not confirmed to ultimately play a role in the pathogenesis of ITAC [4, 5, 7, 8].

While ITACs most frequently involve the nasoethmoidal complex including the posterior ethmoidal cells, the middle turbinate, the posterior-superior septum, the ethmoidal roof and the cribriform plate, involvement of the maxillary sinus is rare [9]. More precisely, Jankowski et al. claimed that ITACs in woodworkers primarily originate from the olfactory cleft, while the nasal septum and turbinate are only secondarily affected [10]. From a prognostic point of view, an advanced tumor stage, sphenoid sinus involvement, orbital, dural or brain infiltration and high-grade histology were established as prognostic factors of poor outcome [7, 11–14].

Recently, tumor budding (TB), a known risk factor in particular for colorectal carcinoma (CRC), but also certain head and neck cancer subtypes [13, 15–20], has been discussed as negative prognostic factor in ITAC [21]. This hypothesis is particularly interesting, since ITAC morphologically, immuno-phenotypically and in part molecularly show a substantial resemblance to CRC [22, 23]. Thus, the aim of this study was to review our ITAC cohort and to determine prognostic factors of outcome, with particular focus on the role of TB.

## Methods

### Study Design

This study received ethical approval from the Ethical Committee of the Canton of Zurich, Switzerland (Approval Number: BASEC 2020-01663). We retrospectively reviewed a consecutive cohort of treatment-naïve ITAC patients, treated at the department of otorhinolaryngology/head and neck surgery at the University Hospital of Zurich (Switzerland), between January 1996 and July 2020. Patients with documented denial to contribute personal health-related data were not included. Included patients from 01/2016 signed a written consent for the use of their material and data. Tumors were staged according to the eight edition of American Joint Committee on Cancer staging system. As described earlier, all patients underwent endoscopic biopsy and exploration of the tumor under general anesthesia [24]. Treatment plans were discussed at our multidisciplinary tumor board.

### Patient Characteristics, Treatment Protocols, Outcome Measures, Follow-Up:

The following patient data and tumor data were collected: age, gender, exposition to occupational carcinogens (wood dust, leather dust), initial clinical classification (cT, cN, cM), tumor stage (stage I–IV) based on clinical and radiological assessment. Treatment protocols consisted of either (1) surgical tumor resection ± postoperative radiation therapy (RT) in intensity-modulated technique (IMRT) or (2) primary radiochemotherapy. Surgical procedures were classified as (1) transnasal endoscopic (including a transnasal-transcribriform approach) resection, (2) cranioendoscopic resection (combination of endoscopic and open approach) or (3) open craniofacial resection [25]. Surgical margins (R0, microscopic tumor (R1), macroscopic tumor (R2) were determined based on the histopathological workup, incorporating specimen margins. Postoperatively, all patients were followed with systematic nasal endoscopy every two months and standardized cross-sectional imaging [26]. Complete remission (CR), recurrence, type of recurrence (local, regional, distant, combination), follow-up (months), state at last follow-up, 3-year and 5-year overall survival (OS, months), and 3-year and 5-year disease-specific survival (DSS, months) as well as 3-year and 5-year disease-free survival (DFS, months) were assessed.

### Histopathological Workup

Histopathological analysis was performed on formalin-fixed, paraffin-embedded ITAC specimens retrieved from the archive of the Department of Molecular Pathology at the University Hospital of Zurich (Switzerland). All cases were reviewed by two blinded, experienced head and neck pathologists (M. D. B.; N. J. R.). According to the Barnes classification, the tumor tissue of all patients was categorized into five morphological subtypes: (1) papillary, (2) colonic, (3) solid, (4) mucinous or (5) mixed and analyzed on hematoxylin and eosin (H&E) staining [27]. Additionally, the presence of stromal and bone infiltration (yes vs. no) were determined. In analogy to CRC, the extent of TB was categorized in one high power field at the hotspot of the invasive front (~ 0.785 mm^2^) using a three-tiered-system as low (0–4 buds), intermediate (5–9 buds) and high (≥ 10 buds) [28]. Of note: patients with either mucinous subtype or patients with no determinable tumor front were not eligible for determination of TB. In selected, especially morphologically poorly differentiated and thus more challenging cases, additional immunohistochemical analyses were performed to undermine ITAC diagnosis. The profile was consistent with previous reports in literature [29, 30]. A subset of cases was additionally tested for expression of DNA mismatch repair (MMR) proteins.

### Immunohistochemical Workup

Commercially available antibodies and institutional internal controls were used on either Leica Bond or Ventana Benchmark automated staining system: Pancytokeratin AE1/AE3, 1:50 [monoclonal], DAKO A/S; Cytokeratin 7, 1:100 [monoclonal] OV-TL 12/30, DAKO A/S; Cytokeratin 20, Prediluted [monoclonal] SP33, Ventana-Roche; CDX2 Protein, prediluted [monoclonal] EPR2764Y, Ventana-Roche; Mouse monoclonal anti-SATB homeobox 2, 1:100 [monoclonal] EP281, BIO-SCIENCE Products AG (Biochemica + Diagnostica); MutL homolog 1, 1:40 [mono-clonal] Es05, Novocastra Laboratories Ltd; Mouse anti-human PMS2, 1:100 [monoclonal], A16-4 PharMingen (Becton Dickinson), MSH2 1:100 [monoclonal] G219-1129, Cell Marque Lifescreen Ltd; MSH6 (SP93), 1:30 [monoclonal] SP93, Cell Marque Lifescreen Ltd.

### Statistical Analysis

The normality of distribution was checked using the Kolmogorov–Smirnov test. Data are either presented as median and interquartile range (IQR) or as mean ± standard deviation (SD), depending on the distribution of data. Differences among the low TB and intermediate/high TB group regarding the distribution of achievement of CR, tumor stage and Barnes subtype were calculated using contingency tables and Fisher’s exact test. For time-to-event-analysis only patients treated in curative intention were included. Regarding the presence of TB, patients were stratified in low-TB (0–4 buds) vs. intermediate/high TB (≥ 5 buds). Disease free survival (DFS) was defined as time from completed primary treatment until relapse any site or death from all causes and included only patients, who achieved CR after initial treatment. Overall survival (OS) was defined from initial diagnosis until death from any cause or last follow-up, while disease specific survival (DSS) was defined from initial diagnosis until death of disease or last follow-up (patients with death of other causes were excluded). Kaplan-Meyer estimates with calculation of log rank statistics were performed to present OS, DSS and DFS and to compare between sub-groups. The end of follow-up was March 2021. A p-value less than 0.05 indicated significance. Statistics used SPSS version 22 (IBM, Armonk, NY).

## Results

### Tumor Characteristics

In total, 31 ITAC patients were included [29 males (93.5%), 2 females (6.5%)]. Mean age at initial diagnosis was 66 years (± 13). In 20/31 patients (64.5%), the past medical history revealed an exposition to wood dust, while in 1/31 patient (3.2%) an exposition to leather dust was observed. Table 1 provides more detailed information on patient and treatment characteristics. Initial tumor stage was stage I in 2/31 patients (6.4%), stage II in 10/31 patients (32.3%), stage III in 8/31 patients (25.8%) and stage IV in 11/31 patients (35.5%).

**Table 1:** Patient and treatment characteristics

	Number of Patients (n, %)
*Initial clinical T category according to clinical and radiological staging (n, %)*	
cT1	2 6.4%)
cT2	10 (32.3%)
cT3	8 (25.8%)
cT4a	4 (12.9%
cT4b	7 (22.6%)
*Initial N category (n, %)*	
cN0	30 (96.8%)
cN+	1 (3.2%)
*Initial M category (n, %)*	
cM0	30 (96.8%)
cM1	1 (3.2%

### Treatment Characteristics, Surgical Margins

Primary treatment protocols consisted of either surgical tumor resection in 30/31 patients (96.8%) or primary radiochemotherapy in 1/31 patient (3.2%). The surgical approach was transnasal endoscopic in 24/30 patients (80%; including a transnasal-transcribriform approach in 18/30 patients), cranioendoscopic in 2/30 patients (6.7%) and craniofacial in 4/30 patients (13.3%). Surgical margins, based on the histopathological workup and intraoperative assessment of the surgeon, were documented as follows: R0 18/30 patients (60%), R1 3/30 patients (10%), R2 4/30 patients (13.3%), not determinable according to surgeon in 5/30 patients (16.7%). Of all 30 surgically treated patients, 20/30 patients (66.7%) underwent adjuvant radiation therapy.

### Outcome Measures: Complete Remission and Recurrences

Overall, CR after primary treatment protocols was achieved in 26/31 patients (83.9%), while 5/31 patients (16.1%) showed progressive/persistent disease. Among all 26 patients with CR, a total of 7 recurrences were observed during the follow-up period. This translates into a recurrence rate of 26.9%, with isolated local recurrence in 6/7 patients and combined loco-regional recurrence with synchronous distant metastases (DM) in 1/7 patient.

### Outcome Measures: Overall Survival, Disease Specific Survival, Disease Free Survival

Among all patients who achieved CR, median DFS was 39 months (IQR 61–71, range 5–183). The 3- and 5-year OS was 83.9% and 78.3% and the 3- and 5-years DSS 83.7% % and 78.5%, respectively. The 3- and 5-year DFS was 77.9% and 69.9%.

### Follow-Up

The median follow-up duration was 39 months (IQR 24–85, range 5–209). At last follow-up, 18/31 patients (58.1%) were alive without disease, 2/31 patients (6.5%) were alive with disease, 6/31 patients (19.4%) died of the disease and 5/31 patients (16.1%) died of other causes.

### Histopathological Workup: Subtypes

**Fig. 1:**
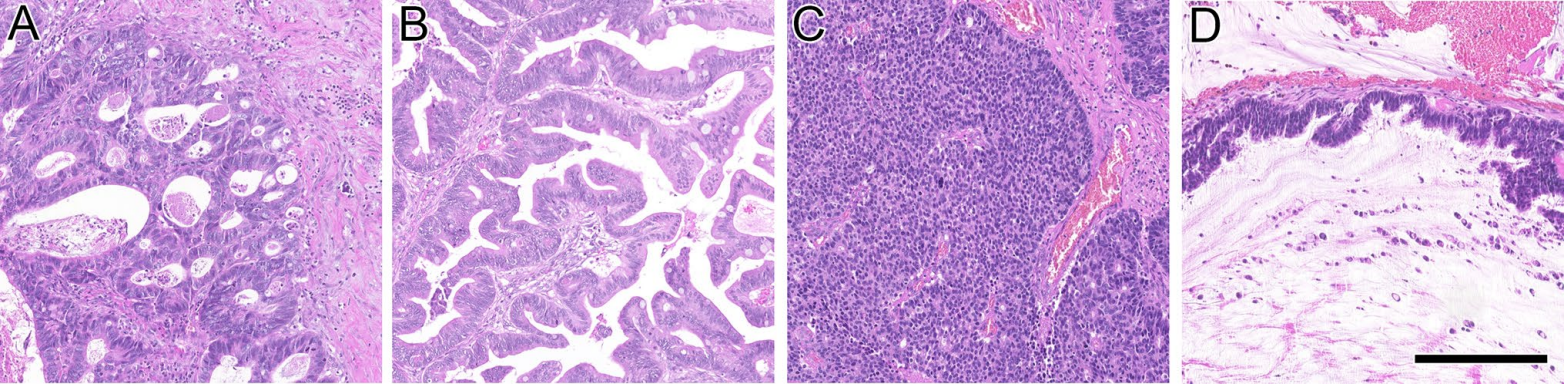
Hematoxylin and eosin (H&E) staining of colonic (**A**), papillary (**B**), solid (**C**) and mucinous (**D**) ITAC. Scale bar 250 µm. ITAC; intestinal-type adenocarcinoma

Barnes subtypes of our cohort was rated as solid in 3/31 patients (9.7%), colonic in 11/31 patients (35.5%), mucinous in 7/31 patients (22.6%), papillary in 6/31 patients (19.4%) and mixed in 4/31 patients (12.8%) (Fig. 1). Histopathological features are summarized in Table 2.

**Table 2:** Summary on histopathological features

Histopathological features	Number of patients (n, %)
*Barnes subtype (n = 31)*	
Solid	3/31 (9.7%)
Colonic	11/31 (35.5%)
Mucinous	7/31 (22.6%)
Papillary	6/31 (19.4%)
Mixed	4/31 (12.8%)
*Infiltration (n = 31)*	
Stromal infiltration	26/31 (83.9%)
Unequivocal bone infiltration	16/31 (51.6%)
*Signet ring cells (n = 31)*	
Present	9/31 (29%)
Absent	22/31 (71%)
*Tumor budding (n = 22)*	
Low	12/22 (54.5%)
Intermediate	6/22 (27.2%)
High	4/22 (18.2%)

### Histopathological Workup: Presence of Infiltration, Presence of Bone Invasion, Signet Ring Cells

Stromal infiltration was present in 26/31 patients (83.9%). Unequivocal bone invasion was seen in 16/31 patients (51.6%). Signet ring cells were present in 9/31 patients (29%). Based on Kaplan Meyer estimates, no significant difference with regard to OS or DSS for presence of (1) signet ring cells, (2) presence of stromal infiltration (yes vs. no) or (3) presence of bone invasion (yes vs. no) was observed.

### Histopathological Workup: Tumor Budding (TB), Stratification of Outcome According to Tumor Budding

**Fig. 2:**
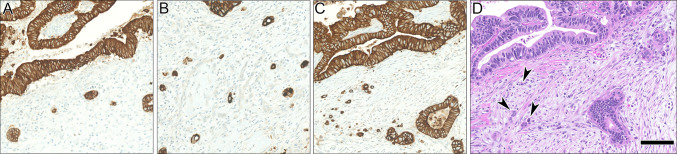
Immunohistochemical pancytokeratin (AE1/AE3) staining indicating low (**A**), intermediate (**B**) and high (**C**) TB. Corresponding HE slide to C, displaying single cells and cell clusters (indicated by arrowheads). Scale bar 100 µm. HE, Hematoxylin and eosin staining; TB, tumor budding

**Fig. 3:**
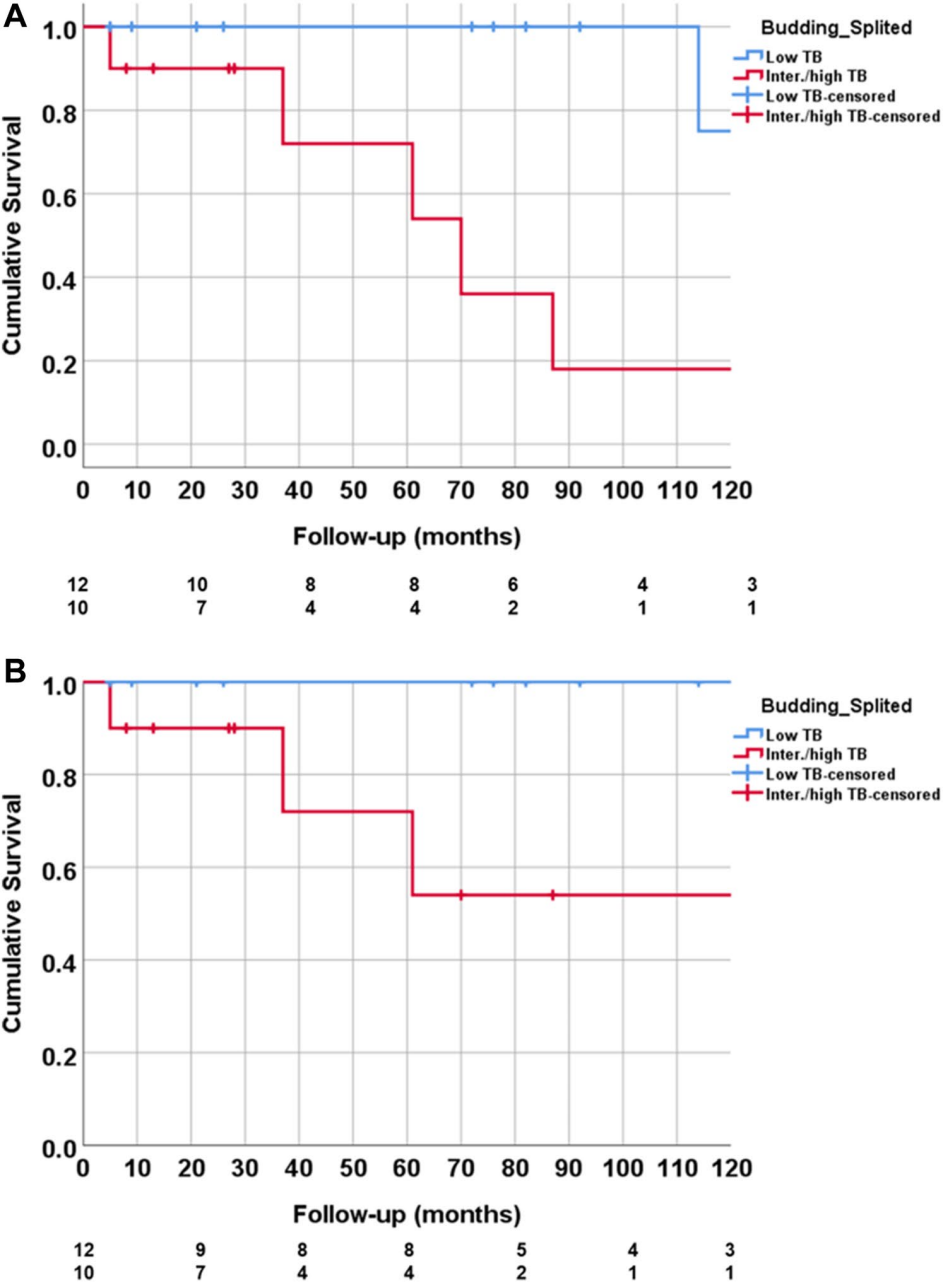
Overall survival (**A**) and DSS (**B**) stratified by low vs. intermediate/high TB. Patients with low TB showed a significant better OS (log-rank test, p = 0.006) and DSS (log-rank test, p = 0.03) and, when compared to intermediate/high TB. DSS, disease-specific survival; TB, tumor budding

Determination of TB was applicable in 22/31 patients (71.0%). Thereof, 12/22 patients (54.5%) revealed a low TB (0–4 buds), 6/22 patients (27.2%) intermediate TB (5–9 buds) and 4/22 (18.2%) patients high TB (≥ 10 buds) (Fig. 2). Patients with low TB showed a significant better DSS (log-rank test, p = 0.03) and OS (log-test, p = 0.006), when compared to intermediate/high TB (Fig. 3). No patient with low TB revealed progressive disease or died of the disease. The distribution of CR achievement among low TB vs. intermediate/high TB was significantly different (Fisher’s exact test; p = 0.03) (Table 3). However, no difference for distribution among tumor stage (Fisher’s exact test; p = 0.09) and Barnes subtype/grading (Fisher’s exact test; p = 0.76) was observed. Disease-free survival was not different between low TB and intermediate/high TB (log-rank test, p = 0.45). Of note: since only one patient with CR developed synchronous regional and DM, no reliable statistics on the distribution of low vs. intermediate/high TB among patients with and without development of metastases could be made.

**Table 3:** Distribution of outcome (complete remission achieved vs. persistent/progressive disease) among low vs. intermediate/high TB. TB, tumor budding

	Low TB	Intermediate /high TB	
Complete remission achieved	12 (54.5%)	6 (27.2%)	18 (81.8%)
Persistent/progressive disease	0 (0%)	4 (18.2%)	4 (18.2%)
	12 (54.5%)	10 (45.45%)	22 (100%)
			Fisher’s exact test p = 0.03

### Histopathological Workup: Immunophenotypical Description

Among 16/31 patients (51.6%) evaluated for Cytokeratin 7, 7/16 patients (43.8%) showed either negative or positive staining, while 2/16 patients (12.5%) revealed only focal positivity. However, all the tested 16 patients were consistently positive for Cytokeratin 20. Overall, 20/31 patients (64.5%) were stained for CDX2, all with a positive result. Furthermore, SATB2 was performed on 9/31 patients (29.05), of which 7/9 patients (77.8%) showed a diffuse positive result, while one patient was negative (11.1%) and another presented with focal positivity (11.1%). In total, 9/31 patients (29.0%) were tested for DNA MMR proteins and showed no sign of loss of any of the markers MLH1, PMS2, MSH2 or MSH6, indicating no evidence of microsatellite instability.

## Discussion

### Main Findings

In this retrospective clinico-pathological study on ITAC patients at a tertiary referral center, we found the presence of intermediate/high TB to be a strong prognostic factor of poor outcome in terms of both, DSS and OS. In contrast to intermediate/high TB, all patients with low TB reached CR after primary treatment protocols and no patient died of disease. However, DFS was not different between low TB and intermediate/high TB patients, indicating that also low TB does not prevent from recurrence. Similar to previous findings and in accordance with CRC, no association between TB and tumor stage or tumor subtype (indicative of grading) was observed.

In our study we confirmed ITAC to mainly originate from the nasoethmoidal complex with a strong predominance for the male gender and incidence peaking in the seventh decade [3, 5, 31]. Overall survival (78.3%), DSS (78.5%) and DFS (69.9%) at 5 years were similar to previous studies, which reported survival rates between 53 and 83%, 82 and 83 and 62 and 74%, respectively [7, 11, 12, 31–34]. With regard to the pattern of recurrence, local recurrence was confirmed to be by far the most common site of treatment failure [12, 32, 35, 36]. From a therapeutic point of view, transnasal endoscopic techniques have evolved as standard-of-reference for most sinonasal malignancies, providing excellent outcomes and decreased morbidity, when compared to external approaches [11, 12, 37]. The role of external approaches remains as an option for selected patients with gross invasion of brain, orbital content or bone [25]. For ITAC in particular, a transnasal endoscopic tumor resection is the preferred approach in most patients, since it can be expanded to a transnasal-craniectomy (transnasal–transcribriform technique), as it was performed in the majority of all surgically treated patients in our cohort (60%) [25]. In case of close proximity or infiltration of the bony or dural anterior skull base, resection of the “ethmoidal box” is pivotal in order to achieve adequate surgical margins [7]. In these patients, dural reconstruction with pedicled flaps or other grafts is mandatory. In terms of adjuvant radiation therapy, there is broad consensus on its necessity in high-grade and/or advanced tumors, while in locally-defined, low-grade tumors with clear surgical margins surgery-only may be justified [7, 12, 14, 38]. Especially for advanced high-grade ITACs as well as patients with exposure to known carcinogens, lifetime clinical and radiological follow up (beyond the often reported 5 years) should be scheduled [7, 39].

As it was shown previously, ITAC and adenocarcinomas of the intestinal tract show to a great extent morphological similarities, as well as overlapping immunohistochemical expression profile, including typically positive staining for CK20, CDX2, villin, MUC2 and SATB2 [22, 23, 40, 41]. As a consequence of this resemblance, TB, a widely accepted concept in CRC, has recently been adapted for ITAC [42]. Tumor budding is to understand as a morphological feature defined as single cells or small cell clusters, so called tumor buds, constituting up to four cells at the invasive front of the tumor or within the tumor mass [21, 43]. For CRC, TB is known to be associated with high and advanced tumor stage, lymphatic and vascular invasion, nodal and distant metastasis and worsening of OS, DFS and recurrence free survival. Furthermore, TB is thought to be part of the epithelial–mesenchymal transition (EMT) spectrum, which allows epithelial cells to lose polarity and cell–cell-adhesions, resulting in migratory and invasive properties, resistance to anoikis/apoptosis and increase extracellular matrix production [21]. Thus, EMT is hypothesized to be one of the drivers of cancer progression [44]. To the best of our knowledge, only Maffeis et al. investigated TB in ITAC and demonstrated that TB is frequent (40%) and associated with worse OS and DFS [21]. Similar to their findings, we found patients with low TB to reveal a favorable prognosis in terms of both, DSS and OS. In comparison, we used the supplementary aid of immunohistochemical pancytokeratin staining, which has been shown to reliably help in identifying tumor buds in CRC [45]. Further it is a highly standardized, cost-effective and widely available special staining. Additionally, we confirmed the presence of TB to be independent from initial tumor stage and Barnes subtype/grading. Interestingly, all patients with low TB reached CR after primary treatment protocols and no patients died of the disease. With regard to DFS, we did not find a significant difference between low and intermediate/high TB. Congruently, recurrences were observed in both groups, however, progressive disease was only seen in the intermediate/high TB group. Based on these findings we hypothesize that low TB does not ultimately protect from development of recurrences. However, patients with intermediate/high TB seem to be at risk for non-treatable disease progression and dead of disease. Interestingly, in our cohort the presence of signet ring cells was not associated with distinct outcomes (OS, DSS), leading us to a more cautious interpretation of this typically negative parameter. With regard to the immunophenotypical description of our cohort and in line with previous findings, no evidence of microsatellite instability was found [40, 46].

### Strengths and Limitations

To the best of our knowledge this is only the second study on the role of TB in ITAC patients [21]. Similar to the study by Maffeis et al., we decided for a validated three-tier-system for TB (low-intermediate-high), depending on the number of buds, however they opted for an overall dichotomous categorization (yes vs. no) [28]. Besides its retrospective design, we acknowledge that our study has some noteworthy limitations. Firstly, although we included all available ITAC patients at our institution, our sample size was rather small, owing to the low incidence of these neoplasms. Consecutively, due to the low absolute numbers of samples and events, no statement on the role of intermediate/high TB for the development of metastases could be made. Secondly, due to natural fragmented histological specimen, identification of the invasive front can be challenging. Thus, the mucinous subtype and patients with no determinable tumor front had to be excluded from this analysis.

### Conclusions

Tumor budding seems to be an independent prognostic factor of worse outcome independent of tumor stage and conventional subtype/grading. In order to promote the implementation into daily clinical practice, additional studies need to corroborate these encouraging data.

## Data Availability

Upon request.

## References

[1] Hoeben A, van de Winkel L, Hoebers F, Kross K, Driessen C, Slootweg P (2016). Intestinal-type sinonasal adenocarcinomas: the road to molecular diagnosis and personalized treatment. Head Neck.

[2] Tripodi D, Quéméner S, Renaudin K, Ferron C, Malard O, Guisle-Marsollier I (2009). Gene expression profiling in sinonasal adenocarcinoma. BMC Med Genomics.

[3] Kılıç S, Samarrai R, Kılıç SS, Mikhael M, Baredes S, Eloy JA (2018). Incidence and survival of sinonasal adenocarcinoma by site and histologic subtype*. Acta Otolaryngol.

[4] Binazzi A, Ferrante P, Marinaccio A (2015). Occupational exposure and sinonasal cancer: a systematic review and meta-analysis. BMC Cancer.

[5] Binazzi A, Corfiati M, Di Marzio D, Cacciatore AM, Zajacovà J, Mensi C (2018). Sinonasal cancer in the Italian national surveillance system: epidemiology, occupation, and public health implications. Am J Ind Med.

[6] Cantu G, Solero CL, Mariani L, Lo Vullo S, Riccio S, Colombo S (2011). Intestinal type adenocarcinoma of the ethmoid sinus in wood and leather workers: a retrospective study of 153 cases. Head Neck.

[7] Rampinelli V, Ferrari M, Nicolai P (2018). Intestinal-type adenocarcinoma of the sinonasal tract: an update. Curr Opin Otolaryngol Head Neck Surg.

[8] Andersson M, Selin F, Järvholm B (2016). Asbestos exposure and the risk of sinonasal cancer. Occup Med (Chic Ill).

[9] Van Gerven L, Jorissen M, Nuyts S, Hermans R, Vander PV (2011). Long-term follow-up of 44 patients with adenocarcinoma of the nasal cavity and sinuses primarily treated with endoscopic resection followed by radiotherapy. Head Neck.

[10] Jankowski R, Georgel T, Vignaud JM, Hemmaoui B, Toussaint B, Graff P (2007). Endoscopic surgery reveals that woodworkers’ adenocarcinomas originate in the olfactory cleft. Rhinology.

[11] Camp S, Van Gerven L, Poorten Vander V, Nuyts S, Hermans R, Hauben E (2016). Long-term follow-up of 123 patients with adenocarcinoma of the sinonasal tract treated with endoscopic resection and postoperative radiation therapy. Head Neck..

[12] Nicolai P, Schreiber A, Bolzoni Villaret A, Lombardi D, Morassi L, Raffetti E (2016). Intestinal type adenocarcinoma of the ethmoid: outcomes of a treatment regimen based on endoscopic surgery with or without radiotherapy. Head Neck.

[13] Grigore A, Jolly M, Jia D, Farach-Carson M, Levine H (2016). Tumor budding: the name is EMT. Partial EMT J Clin Med.

[14] Choussy O, Ferron C, Védrine PO, Toussaint B, Liétin B, Marandas P (2010). Role of radiotherapy in the treatment of nasoethmoidal adenocarcinoma. Arch Otolaryngol Head Neck Surg.

[15] Almangush A, Pirinen M, Heikkinen I, Mäkitie AA, Salo T, Leivo I (2018). Tumour budding in oral squamous cell carcinoma: a meta-analysis. Br J Cancer.

[16] Karamitopoulou E, Zlobec I, Born D, Kondi-Pafiti A, Lykoudis P, Mellou A (2013). Tumour budding is a strong and independent prognostic factor in pancreatic cancer. Eur J Cancer.

[17] Mäkitie AA, Almangush A, Rodrigo JP, Ferlito A, Leivo I (2019). Hallmarks of cancer: Tumor budding as a sign of invasion and metastasis in head and neck cancer. Head Neck.

[18] Cappellesso R, Luchini C, Veronese N, Lo Mele M, Rosa-Rizzotto E, Guido E (2017). Tumor budding as a risk factor for nodal metastasis in pT1 colorectal cancers: a meta-analysis. Hum Pathol.

[19] Petrelli F, Pezzica E, Cabiddu M, Coinu A, Borgonovo K, Ghilardi M (2015). Tumour budding and survival in Stage II colorectal cancer: a systematic review and pooled analysis. J Gastrointest Cancer.

[20] Maffeis V, Nicolè L, Cappellesso R (2019). RAS, Cellular plasticity, and tumor budding in colorectal cancer. Front Oncol.

[21] Maffeis V, Cappellesso R, Galuppini F, Guzzardo V, Zanon A, Cazzador D (2020). Tumor budding is an adverse prognostic marker in intestinal-type sinonasal adenocarcinoma and seems to be unrelated to epithelial-mesenchymal transition. Virchows Arch.

[22] Franchi A, Innocenti DRD, Palomba A, Miligi L, Paiar F, Franzese C (2014). Low prevalence of K-RAS, EGF-R and BRAF mutations in sinonasal adenocarcinomas. Implications for anti-EGFR treatments. Pathol Oncol Res.

[23] Franchi A, Massi D, Palomba A, Biancalani M, Santucci M (2004). CDX-2, cytokeratin 7 and cytokeratin 20 immunohistochemical expression in the differential diagnosis of primary adenocarcinomas of the sinonasal tract. Virchows Arch.

[24] Meerwein CM, Pazahr S, Soyka MB, Hüllner MW, Holzmann D (2020). Diagnostic accuracy of computed tomography and magnetic resonance imaging compared to surgical exploration for anterior skull base and medial orbital wall infiltration in advanced sinonasal tumors. Head Neck.

[25] Castelnuovo P, Turri-Zanoni M, Battaglia P, Antognoni P, Bossi P, Locatelli D (2016). Sinonasal malignancies of anterior skull base: histology-driven treatment strategies. Otolaryngol Clin N Am.

[26] Meerwein CM, Pangalu A, Pazahr S, Epprecht L, Soyka MB, Holzmann D (2021). The Zurich magnetic resonance imaging protocol for standardized staging and restaging of sinonasal tumours*. Rhinol Online.

[27] Barnes L (1986). Intestinal-type adenocarcinoma of the nasal cavity and paranasal sinuses. Am J Surg Pathol..

[28] Lugli A, Kirsch R, Ajioka Y, Bosman F, Cathomas G, Dawson H (2017). Recommendations for reporting tumor budding in colorectal cancer based on the International Tumor Budding Consensus Conference (ITBCC) 2016. Mod Pathol.

[29] Kennedy MT, Jordan RCK, Berean KW, Perez-Ordoñez B (2004). Expression pattern of CK7, CK20, CDX-2, and villin in intestinal-type sinonasal adenocarcinoma. J Clin Pathol.

[30] Cathro HP, Mills SE (2004). Immunophenotypic differences between intestinal-type and low-grade papillary sinonasal adenocarcinomas: an immunohistochemical study of 22 cases utilizing CDX2 and MUC2. Am J Surg Pathol.

[31] D’Aguillo CM, Kanumuri VV, Khan MN, Sanghvi S, Patel NR, Baredes S (2014). Demographics and survival trends of sinonasal adenocarcinoma from 1973 to 2009. Int Forum Allergy Rhinol.

[32] Vergez S, Du Mayne MD, Coste A, Gallet P, Jankowski R, Dufour X (2014). Multicenter study to assess endoscopic resection of 159 sinonasal adenocarcinomas. Ann Surg Oncol.

[33] Bogaerts S, Poorten Vander V, Nuyts S, Van Den Bogaert W, Jorisser M (2008). Results of endoscopic resection followed by radiotherapy for primarily diagnosed adenocarcinomas of the paranasal sinuses. Head Neck.

[34] Parasher AK, Kuan EC, John MAS, Tajudeen BA, Adappa ND (2018). What is the appropriate timing for endoscopic and radiographic surveillance following treatment for sinonasal malignancies?. Laryngoscope.

[35] Donhuijsen K, Kollecker I, Petersen P, Gaßler N, Schulze J, Schroeder HG (2016). Metastatic behaviour of sinonasal adenocarcinomas of the intestinal type (ITAC). Eur Arch Oto-Rhino-Laryngology.

[36] Meerwein CM, Balermpas P, Vital DG, Broglie MA, Soyka MB, Holzmann D (2021). The role of regional disease and patterns of treatment failure in primary sinonasal malignancies. Am J Rhinol Allergy.

[37] Meccariello G, Deganello A, Choussy O, Gallo O, Vitali D, De Raucourt D (2016). Endoscopic nasal versus open approach for the management of sinonasal adenocarcinoma: a pooled-analysis of 1826 patients. Head Neck.

[38] Turri-Zanoni M, Battaglia P, Lambertoni A, Giovannardi M, Schreiber A, Volpi L (2015). Treatment strategies for primary early-stage sinonasal adenocarcinoma: a retrospective bi-institutional case-control study. J Surg Oncol.

[39] Gallet P, Nguyen DT, Russel A, Jankowski R, Vigouroux C, Rumeau C (2018). Intestinal and non-intestinal nasal cavity adenocarcinoma: Impact of wood dust exposure. Eur Ann Otorhinolaryngol Head Neck Dis.

[40] Skalova A, Sar A, Laco J, Metelkova A, Miesbauerova M, Steiner P (2018). The role of SATB2 as a diagnostic marker of sinonasal intestinal-type adenocarcinoma. Appl Immunohistochem Mol Morphol..

[41] Berg KB, Schaeffer DF (2017). SATB2 as an immunohistochemical marker for colorectal adenocarcinoma a concise review of benefits and pitfalls. Arch Pathol Lab Med.

[42] WHO Classification of Tumours Editorial Board. Digestive System Tumours (WHO classification of tumours series, 5th edition, vol. 1). Lyon (France): International Agency for Research on Cancer. 2019.

[43] Dawson H, Galuppini F, Träger P, Berger MD, Studer P, Brügger L (2019). Validation of the International Tumor Budding Consensus Conference 2016 recommendations on tumor budding in stage I-IV colorectal cancer. Hum Pathol.

[44] Kalluri R, Weinberg RA (2009). The basics of epithelial-mesenchymal transition. J Clin Invest.

[45] Koelzer VH, Assarzadegan N, Dawson H, Mitrovic B, Grin A, Messenger DE (2017). Cytokeratin-based assessment of tumour budding in colorectal cancer: analysis in stage II patients and prospective diagnostic experience. J Pathol Clin Res.

[46] Perez-Ordonez B, Huynh NN, Berean KW, Jordan RCK (2004). Expression of mismatch repair proteins, β catenin, and E cadherin in intestinal-type sinonasal adenocarcinoma. J Clin Pathol.

